# Secure Utilization of Beacons and UAVs in Emergency Response Systems for Building Fire Hazard

**DOI:** 10.3390/s17102200

**Published:** 2017-09-25

**Authors:** Seung-Hyun Seo, Jung-In Choi, Jinseok Song

**Affiliations:** The Division of Electrical Engineering, Hanyang University, ERICA Campus, Gyeonggi-do, Ansan 15588, Korea; peach0206@hanyang.ac.kr (J.-I.C.); bbtam99@naver.com (J.S.)

**Keywords:** sensors and beacons, unmanned aerial vehicles, monitoring and emergency response systems, Internet of Things, security

## Abstract

An intelligent emergency system for hazard monitoring and building evacuation is a very important application area in Internet of Things (IoT) technology. Through the use of smart sensors, such a system can provide more vital and reliable information to first-responders and also reduce the incidents of false alarms. Several smart monitoring and warning systems do already exist, though they exhibit key weaknesses such as a limited monitoring coverage and security, which have not yet been sufficiently addressed. In this paper, we propose a monitoring and emergency response method for buildings by utilizing beacons and Unmanned Aerial Vehicles (UAVs) on an IoT security platform. In order to demonstrate the practicability of our method, we also implement a proof of concept prototype, which we call the UAV-EMOR (UAV-assisted Emergency Monitoring and Response) system. Our UAV-EMOR system provides the following novel features: (1) secure communications between UAVs, smart sensors, the control server and a smartphone app for security managers; (2) enhanced coordination between smart sensors and indoor/outdoor UAVs to expand real-time monitoring coverage; and (3) beacon-aided rescue and building evacuation.

## 1. Introduction

Fire detection and corresponding safety systems like monitoring and emergency response systems are crucial aspects of any building management system, and billions of dollars are spent annually on the installation and maintenance of such systems [1]. However, with recent advances in IoT technology, intelligent emergency systems using smart sensors offer opportunities to accomplish these tasks more efficiently and economically. Smart sensors can often anticipate emergencies even before they happen, and when emergencies do occur, such sensors can provide more complete and reliable information to building management and emergency personnel. This allows building occupants to be evacuated more quickly and safely than is possible when using conventional systems. For these reasons, it is becoming standard for all new buildings to be equipped with wireless sensors that function as part of the overall building management system. In fact, many proposed alert and response systems already incorporate wireless sensors in their designs [2,3,4,5,6]. However, in these proposals [2,3,4,5,6], sensors are used only in the monitoring of interior locations, which leaves building exteriors insufficiently monitored. Furthermore, since such systems mostly use fixed cameras like closed-circuit television(CCTV) for monitoring, even many areas inside buildings remain vulnerable. Unmanned Aerial Vehicles (UAVs), also known as drones, are another recently-emerging technology that is being used more frequently for monitoring and surveillance in many common public sectors. Specifically, camera-equipped drones have been widely used for visual monitoring in public safety, traffic management and disaster monitoring.

Moreover, UAVs can be used to support search and rescue operations and perform operations that are hard to execute by human operators, at low operating costs. When UAVs are deployed in a disaster area, they can collect evidence of the presence of a victim and report the collected data to a rescue team. Some monitoring and warning and search and rescue systems have already proposed the use of UAVs [7,8,9,10,11,12,13,14,15,16,17], but since most of those systems focus on natural disasters, they are not directly applicable to the monitoring of buildings. Another important weakness of those systems is that they do not account for the secure transmission of data between sensors/drones and control servers. This leaves such systems highly vulnerable to malicious attacks in which people outside of the system could easily alter the data that are being transmitted or even take direct control of various aspects of the system.

In order to address these several constraints, in this paper, we propose a method of property monitoring and emergency response by utilizing beacons and UAVs on an IoT open standard platform such as the AllJoyn platform [18]. We also present a proof of concept prototype, called the UAV-EMOR (UAV-assisted Emergency Monitoring and Response) system. Our UAV-EMOR system offers the following three novel features.

First, our UAV-EMOR system supports end-to-end secure communication between all components of the system, including UAVs, smart sensors, the control server and a smartphone app for security managers. Unfortunately, one aspect of wireless network-based monitoring and emergency response systems that has not been sufficiently addressed thus far is data security. Systems that use wireless networks are vulnerable to external manipulation, impersonation and eavesdropping, etc. In our system, as well, all entities are connected to the control server via wireless networks, and in order to provide secure data transmission, we incorporate the standard authenticated key exchange protocol supported by the Security 2.0 specification of the AllJoyn IoT platform [18]. Since a security manager app is used to remotely monitor the interior and exterior of the building and to access the emergency response systems, it is vital that only a legitimate security manager should be capable of accessing the app and communicating with the emergency response system. To ensure this is the case, we use a bio-metric authentication method using fingerprint identification.

Another key aspect of our system is the integration of UAVs. Our UAV-EMOR system supports enhanced coordination between smart sensors and indoor/outdoor drones in order to expand real-time monitoring coverage. The existing research on UAV-assisted monitoring and warning systems mainly focuses on environmental monitoring and emergency response for natural disasters [7,8,9,10,11,12,13,14]. In our work, we introduce a more dynamic monitoring approach, which uses adaptive indoor/outdoor UAVs to expand monitoring coverage for buildings and territory around them. Indoor UAVs are assigned to each floor of a building, while outdoor UAVs are used to monitor the exterior. This allows our system to monitor the blind spots that the fixed CCTV of conventional systems cannot capture.

Unmanned all-terrain vehicle(ATV) can be utilized for monitoring instead of indoor drones [19]. However, we are only considering devices that can be operated for indoor monitoring without any problem even in an emergency situation. In a disaster situation where buildings are collapsing, ATVs are difficult to get into the room and to move in situations where users are evacuating quickly. ATVs can have visibility issues that the UAV would not have since the UAV would be able to move above and around the flames. ATVs would have benefits in other ways, but their size makes them difficult to stock many in the building itself. More UAVs could be kept on site. While the battery may become an issue, the UAV only has to fly for a short time to collect data and would not necessarily run out of battery. Plus, a large enough supply of smaller UAVs could offset the battery issue. Therefore, an emergency response method using indoor UAVs is more effective than ATVs.

Finally, our UAV-EMOR system provides beacon-aided rescue and building evacuation. There is a distinct need to improve emergency response methods so building occupants can be more efficiently and safely evacuated during emergency situations. There are currently several approaches [20,21,22] that attempt to track the location of individuals inside of buildings. However, they are not precise enough to be practicable or they require an active response from a user, which may not be possible during certain types of emergencies. For example, some systems use the global positioning system(GPS) of users’ smartphones for location, but GPS-based location information cannot be calculated accurately indoors [23]. Other systems use a variety of sensors like ultrasonic, near-field communication and radio frequency signals, but those systems are overly dependent on battery power and require active interaction from users [20,21,22]. Fixed location beacons are also in use and require very little battery power, though they suffer from the same issue as sensors, as they are dependent on user interaction [24,25]. In our approach, we take advantage of the beacon’s low battery consumption and increase its effectiveness by assigning an individual beacon to every person inside a building. This is accomplished by incorporating beacons into smart ID cards that are issued to all building occupants. Using Bluetooth technology, such beacons constantly transmit the unique ID and real-time location of the wearer to all smart sensors and drones. The beacons have no on/off function, so there is never any danger of accidentally disabling the device; nor do they require active input from the user, so they can be used to locate users who are perhaps too badly injured to activate a device or who are unconscious. Furthermore, since beacon signals can transmit through building walls, users can be located even inside of collapsed buildings.

The remainder of this paper is organized as follows: In Section 2, we discuss related works and provide a comparison of our approach with those recent works. In Section 3, we introduce the main components of the UAV-EMOR system and present system protocols. In Section 4, we describe the prototype of our UAV-EMOR system and use scenarios. In Section 5, we evaluate communication and computation overhead for the performance of our system protocols and also the evaluate position accuracy of our UAV-EMOR system. Lastly, in Section 6, we outline our conclusions.

## 2. Related Works and Backgrounds

In this section, we first explore related works in the field of emergency alert and response systems. Then, we discuss several related works for warning systems that incorporate UAVs. We will also look at works that address how to incorporate sensor-based monitoring systems into buildings that were not designed with this purpose in mind. Table 1 summarizes the comparison of recent related works.

### 2.1. Emergency Alert and Response System

Conventional emergency and evacuation systems do not provide information about the context of an emergency, nor about the profile of each person in the building. To address these weaknesses, there is a growing interest in alert and response systems using smart sensors that are tailored to the specifics of an emergency situation. Aedo, I. et al. [2] propose a set of criteria related to user profiles, available technologies, kinds of emergency and contextual circumstances for an effective notification and evacuation. The authors present a mechanism that provides personalized alert notifications and evacuation routes to users with smartphones. However, their mechanism cannot automatically identify the building occupant’s location. To get the personalized evacuation route, occupants should send their location data to the emergency management server. This system has a disadvantage in that it cannot be used if a part of the building collapses and the occupant cannot grasp the current location.

Liu, J.W.S. et al. [3] propose a data model and architecture of indoor emergency evacuation systems for large public buildings. The authors also present an intelligent indoor emergency evacuation system that utilizes the Building and environment Data-based Indoor Positioning System (BeDIPS) [26] to support indoor positioning service. However, BeDIPS requires installing location beacons in a building, and in the current version of BeDIPS, each location beacon only provides a one-step navigation instruction to the nearest exit. Thus, Liu, J.W.S. et al.’s system could not support a personalized evacuation route for each occupant.

Wu, Z.Y. et al. [4] propose a framework for an intelligent evacuation guidance system for large buildings. They combine fire detection, video surveillance, a mobile terminal and passive RFID tags to create an evacuation guidance system. During regular monitoring, their system notes temperature and atmospheric changes, setting off an alarm when appropriate. During an actual emergency situation, the system monitors population density inside the building, the evacuation status of all entrances and the road capacity outside the building. Furthermore, their system can guide individuals from their specific locations in the building to appropriate exits by sending out evacuation instructions. However, their RFID-based evacuation system requires active input from the occupants to get the evacuation instructions.

Zeng, Y. et al. [5] propose an emergency routing scheme for wireless sensor networks that is designed specifically for building fire emergency. Their scheme is adaptable to several different scenarios and the routing hole problems. Each sensor contains data about its own ID, power status and state and automatically changes its state between safe, low safe, fire and unsafe. Lin, C.Y. et al. [6] propose an active disaster response system for earthquakes, which automatically engages when a natural disaster occurs. Their system consists of two main parts, an emergency broadcast system and an active disaster response system. In the emergency broadcast system, the weather center sends warning messages to embedded boards. Then, the active disaster response system automatically performs specified actions, like cutting off power and gas. During a natural disaster, this system configures embedded boards to construct and maintain a temporary network for sending emergency messages. Using this network, the rescue center can integrate data and enable rescue workers to provide aid where it is needed.

Unlike these previous works, our UAVs-EMOR system supports both indoor and outdoor monitoring by incorporating UAVs and automatically identifies the occupant’s location by using beacon technology. Therefore, it can provide evacuation information to occupants.

### 2.2. UAV-Based Warning System

UAVs deliver timely disaster warnings to public officers and support rescue and recovery operations. Due to their ability to fly autonomously and rapidly acquire sensing data on emergency areas that are inaccessible to humans, UAVs have become a viral candidate platform for emergency monitoring and response systems. Drones are being used by organizations such as customs control, the coast guard, environmental agencies and private businesses for a variety of purposes including safety systems in the gas and chemical industry, fire detection, tactical surveillance, search and rescue, environmental monitoring, and many more [8,12,27,28,29].

Choi, K. et al. [7] propose a monitoring system for emergency responses. Their system consists of both an aerial and a ground component. The aerial aspect includes a UAV platform, sensors and supporting modules, while the ground aspect includes vehicles and a receiving and processing system. The aerial component receives sensor data from the environment and transmits it to the ground component. Sensor data and control commands are transmitted automatically in real time.

Chen, D. et al. [9] propose a dynamic routing protocol, a method for managing mobile nodes to enable reliable, real-time data transmission. In [9], to support early warning, UAVs monitor the area and collect sensor data, transferring it to the data center for analysis. Frigerio, S. et al. [10] propose a web-based platform for automatic and continuous monitoring of the Rotolon catchment in the Italian Alps for potential landslides. Ueyama, J. et al. [11] propose the use of UAVs to make their wireless sensor network more resilient to failures for river monitoring.

Most warning systems based on UAVs focus on natural disasters, and such systems are unsuitable for application in security monitoring. In the IoT environment, each individual device and sensor must contain a security module to protect it from malicious interference. Indeed, in an emergency response system for a building, it is most important to prevent malicious attacks on the communications network. Therefore, we customize security modules from the AllJoyn Security Platform to secure every component of our system.

### 2.3. Incorporating Sensor-Based Monitoring Systems into Existing Buildings

The term “smart building” describes a suite of technologies used to make the design, construction and operation of buildings more efficient and is applicable to both existing and newly-built properties [30]. The smart building in the future will adjust in every situation to provide personalized service to every individual inside the building. To do this, the building must have the ability to sense and to interpret situations automatically. Buildings without smart technology cannot transmit environmental information in real time because they do not incorporate network-connected sensors. However, reconstructing a building to make it “smart” would be an inefficient waste of both time and financial resources.

For energy savings in commercial buildings, Weng, T. et al. [31] have presented a design for converting a conventional building into a smart building. They have developed several mechanisms for activating the HVAC system, IT equipment and lighting depending on occupancy and usage. For monitoring, they attach sensors to the building and install building management systems. They estimate that the cost of converting a building to make it “smart” is offset by the energy savings it produces. Osello, A. et al. [1] also propose using middleware to reduce energy in existing buildings. They use software to monitor and control the lighting and HVAC services. They also design the context framework using an ontology-based knowledge repository and a rule-based context recognition system. Whereas existing smart building systems focus primarily on energy-reduction, our system provides a cost-effective, smart emergency alert and response network that provides situationally-specific and personalized service for users.

## 3. UAV-Assisted Emergency Monitoring and Response System

In this section, we briefly describe an overview of our UAV-assisted Emergency Monitoring and Response (UAV-EMOR) system for buildings. We then present each of the processes of which our system is composed: (1) day-to-day monitoring; (2) abnormal situation response; (3) emergency response.

### 3.1. Overview of UAV-EMOR System

**Figure 1:**
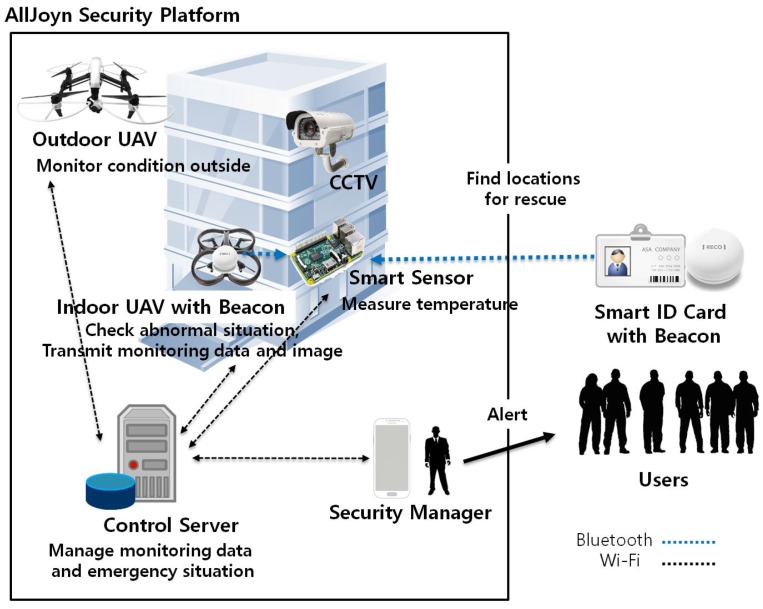
The overview of our UAV-assisted emergency monitoring and response system.

There are four novel functionalities of our UAV-EMOR system: (1) provide secure communications between UAVs, smart sensors, a control server and the security manager’s smartphone app, which is based on the AllJoyn security platform; (2) support real-time monitoring using smart sensors; (3) support active response using indoor/outdoor UAVs; and (4) support beacon-aided rescue. Figure 1 provides an overview of our UAV-EMOR system. The system consists of six entities: smart sensors, indoor/outdoor monitoring UAVs (Unmanned Aerial Vehicles), a security manager, a control server, users and CCTV. Indoor and outdoor UAVs periodically follow designated routes, monitor conditions inside and outside of the building while interfacing with smart sensors that have been placed in each room. Each smart sensor checks the surrounding temperature and notes potentially abnormal situations inside the room. Each UAV transmits its monitoring data and images directly to the control server, where a building security manager can monitor the images at any time. CCTV is still used to monitor the space for potential dangers, like fire, and stores those recordings on the server. However, the indoor UAVs on each floor can focus on blind spots that CCTV cannot see.

The indoor UAV only monitors when it is given a task, but UAVs currently in commercial use have an average of about 30 min–1 h of battery life, which is sufficient for most tasks. So after use, they must be recharged. To solve this battery issue, we assume that an indoor UAV usually stays in a specific landing spot on each floor. The indoor UAV can remain charged at the landing spot until it receives the command from the control server.

**Figure 2:**
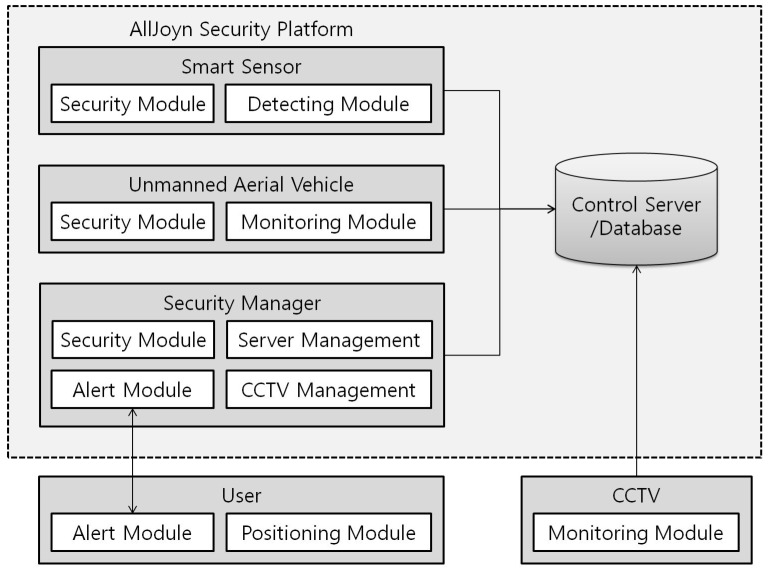
The Main components of the UAV-EMOR system.

The Bluetooth beacons are attached to or embedded in the indoor UAVs and the users’ smart ID cards for indoor location recognition. The indoor UAV’s beacon transmits the ID of the indoor UAV to the smart sensor. Only authorized UAVs can get entry into the room. The beacons in the smart ID cards transmit a signal to the smart sensor in an emergency situation so that the smart sensor determines the number of users and the users’ information in each room. The smart sensors, UAVs and security manager’s smartphone are all connected to the control server via the wireless network. We use the AllJoyn security platform in order to provide secure communications among the various components of our system. Figure 2 shows the main components of UAV-EMOR system.

### 3.2. AllJoyn Security Platform for UAV-EMOR System

The AllJoyn system supports a security framework for IoT applications to authenticate each other and send encrypted data between them. Since AllJoyn applications can be installed on the IoT devices, it can be utilized to provide end-to-end application level security between IoT devices, and thus authentication and data encryption are done at the application layer. All of the logic for authentication and encryption except the Auth is implemented in the AllJoyn core library. The Auth Listener is a callback function implemented by the application to provide auth credentials (e.g., PIN or password) or verify auth credentials (e.g., verify the certificate chain in the case of ALLJOYN_ECDHE_ECDSA). The security module manages a key store to save authentication and encryption keys. The AllJoyn security platform makes use of the ECDHE (Elliptic Curve based Diffie–Hellman Key Exchange) [32] algorithm for generating session keys. Furthermore, it utilizes the Simple Authentication and Security Layer (SASL) [33] security framework for authentication and key exchange. It supports the following authentication mechanisms for app-to-app level authentication:

- ALLJOYN_SRP_KEYX: Secure Remote Password (SRP) key exchange.
- ALLJOYN_SRP_LOGON: Secure Remote Password (SRP) log on with username and password.
- ALLJOYN_ECDHE_NULL: Elliptic Curve Diffie–Hellman (ephemeral) key exchange with no authentication.
- ALLJOYN_ECDHE_PSK: Elliptic Curve Diffie–Hellman (ephemeral) key exchange authenticated with a pre-shared key (PSK).
- ALLJOYN_ECDHE_ECDSA: Elliptic Curve Diffie–Hellman (ephemeral) key exchange authenticated with an X.509 ECDSA certificate.

**Figure 3:**
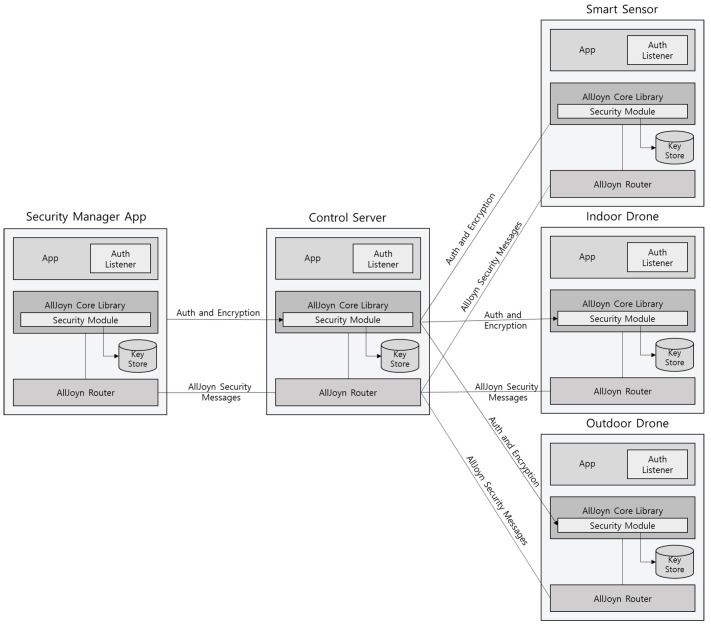
The security architecture of our system.

We applied the ALLJOYN_ECDHE_PSK mechanism to support authentication and secure communication between all components. Figure 3 shows an overview of the security architecture of our UAV-EMOR system. This ALLJOYN_ECDHE_PSK mechanism provides the pre-shared key-based authenticated ECDHE [32] to share the communication session encryption key between all components. In the setup phase, the control server generates a Pre-shared Secret Key (PSK) with each of the UAVs, the security manager’s smartphone and the smart sensors. Each component shares a unique PSK with the control server. In order to generate a session encryption key, the pre-shared key-based authenticated ECDHE protocol should be performed between all components.

**Figure 4:**
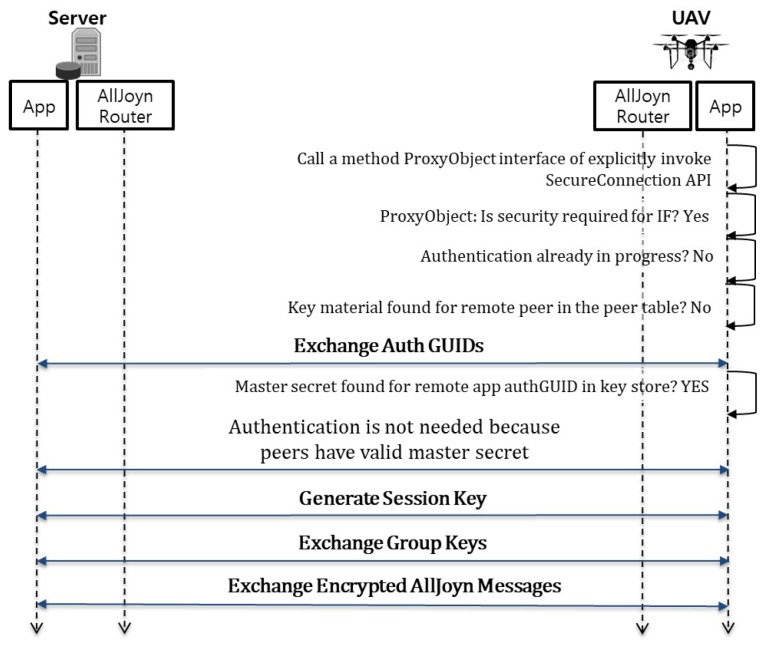
The end to end security flow of our UAV-EMOR system.

Since all components such as UAVs, smart sensors, etc., have already been authenticated, a PSK is found in the key store. Therefore, the UAV can directly execute the next step of generating a session key and/or a group key. Each component may generate a session key with the control server by using the ECDHE algorithm based on the pre-shared key authentication. Once the session key is generated, it is used along with the AES (Advanced Encryption Standard) [34] algorithm to encrypt communication messages. This is accomplished by installing the AllJoyn security platform on the control server and the security manager’s smartphone, as well as on all of the UAVs and smart sensors. Figure 4 shows the high-level message flow of the end to end security communication between the control server and the UAV as an example. This flow consists of following four steps:

- Exchange Auth GUIDs (Authentication Group User Identifications): In this step, two peers such as the control server and the UAV exchange Auth GUIDs. The Auth GUID is used to see if the pre-shared key is present for that Auth GUID in the key store. If no pre-shared key is found, the two peers have not authenticated each other. Therefore, the two peers should execute the next app-to-app authentication step.
- App-to-app authentication: In this step, two peers perform the authentication mechanism supported by the AllJoyn platform. At the end of this step, the two peers have authenticated each other and share a common pre-shared key.
- Generate a session key: In this step, two peers generate a session key for encrypting communication messages between them. The session key is generated independently by both peers based on the pre-shared key. A group key is also generated when the first session key is generated.
- Exchange group keys: In this step, two peers exchange their own group keys with each other via an encrypted AllJoyn message. The group key is used to encrypt the session multicast and broadcast signals. At the end of this step, the two peers share group keys to decrypt secure broadcast signals received from each other.

### 3.3. System Protocols

**Figure 5:**
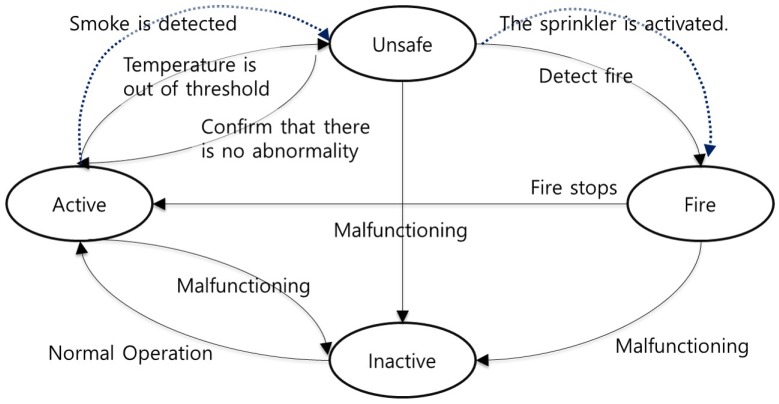
State diagram of a smart sensor.

In our UAV-EMOR system, a smart sensor indicates one of four states: (1) “Active”: indicates that the sensor is functioning correctly; (2) “Unsafe”: indicates when the local temperature is beyond an established threshold; (3) “Fire”: indicates when a fire has been detected; (4) “Inactive”: indicates that the sensor is no longer functioning. A smart sensor automatically indicates these states, according to the settings chosen by the system administrator, as shown in Figure 5.

- Active: When a smart sensor is functioning correctly or if it detects that a fire has ended, it indicates this by sending an “Active” message to the server.
- Unsafe: If a smart sensor detects an abnormal temperature or the smoke detection system detects smoke , it is indicated with the message “Unsafe”. The manager receives the abnormal temperature data or the smoke data and confirms whether there is an abnormality or not.
- Fire: If a manager confirms that there is a fire, the smart sensor enters the “Fire” state. When the control server receives the “Fire” message, it guides UAVs to observe the location of the smart sensor where the fire has been detected and the sprinkler is activated Furthermore, the control server sends an emergency message to everyone who is connected to the system. Via beacons, the smart sensors collect user positions and send them to the control server and manager.
- Inactive: This status indicates that a smart sensor is no longer functioning correctly due to error, loss of power, etc. In such a situation, the smart sensor notifies the control server of its “Inactive” state.

Algorithm 1 shows the overall emergency management algorithm of the control server.

**Algorithm 1:** Management Algorithm of Control Server   
State : Active, InActive, Unsafe, Fire   Tt : Temperature value in time T   Tm : Normal threshold of temperature
   IPhoto : Photo taken by an indoor drone   OPhoto : Photo taken by an outdoor drone   CCTVimage : image taken by CCTV   Monitoring Group : Indoor Drone, Outdoor Drone, CCTV, Smart Sensor  **1** Connect to Smart Sensor  **2**
**if**
*Smart Sensor == InActive*
**then**  **3** Transmit “InActive” message to Security Manager  **4**
**end**  **5**
**while**
*Smart Sensor == Active*
**do**  **6** Receive Tt from Smart Sensor  **7** **if**
*The temperature change == True*
**then**  **8**  Save Tt to DB  **9**  Transmit Warning Message to Security Manager **10**  **if**
*Tt < Tm*
**then** **11**   Receive “Safe” message from Security Manager **12**   Run Normal Operation of Day-to-Day Monitoring **13**  **end** **14**  **if**
*Tt >= Tm*
**then** **15**   State = UnSafe **16**   Broadcast “UnSafe” message to Security Manager and Monitoring Group **17**   **while**
*State == Unsafe of Fire*
**do** **18**    Run “Abnormal Response” operation **19**    Run the fire detection system (or smoke detection system) **20**    Update IPhoto in DB **21**    Update OPhoto in DB **22**    Update CCTVimage in DB **23**    Receive Tt from Smart sensors **24**    Receive State and location from Security Manager **25**    **if**
*State == Fire*
**then** **26**     Broadcast “Fire” message to Users and Monitoring Group **27**     Run “Emergency Response” operation **28**     Run the sprinkler system **29**    **end** **30**   **end** **31**  **end** **32** **end** **33**
**end**

#### 3.3.1. Day to Day Monitoring

The temperature in each room is constantly monitored by a smart sensor attached near the entrance of the room. To maximize efficiency, a smart sensor only transmits a temperature value to the control server if the temperature value changes. The server stores the received temperature value and checks whether it is over a certain range of temperature. If the temperature value goes beyond a certain range, the server broadcasts the message “Unsafe” to an indoor drone located in the same floor, a security manager and all smart sensors in the building. The drone is then directed to the location of the smart sensor that sent the high temperature value to take a photo and video of the zone. Then, the drone sends the photo and video to the control server. The security manager can access the server and monitor these images to determine whether or not the situation is abnormal. If it is an abnormal situation, the “Abnormal Situation Response Process” is engaged. If the situation is safe, then the server broadcasts the message “Safe” to the indoor drone, smart sensors and a security manager’s smartphone. In the absence of any “Unsafe” messages, all parts of the system continue with their regular daily monitoring duties.

#### 3.3.2. Abnormal Situation Response

When a smart sensor detects that the temperature has climbed above a set threshold, the server immediately sends a message to the drones and security managers, informing them of the location where the abnormal situation has been detected, as well as the present temperature at that location. Upon receiving that message, the outdoor drone moves to the location under question, where it takes photos of the area and immediately sends them back to the server. Concurrently, inside the building on the floor where the abnormal situation was detected, an indoor drone moves to the location. Since the temperature sensor is located at the entrance way to the room, it cannot determine whether the abnormal temperature has occurred inside the room or outside in the hallway. Therefore, when the drone arrives at the location of the abnormal temperature change, it first photographs the hallway area just outside the room and sends that data to the server. Then, it moves into the room to take photographs there. To enter the room, the drone requires authentication, and it is outfitted with a beacon sensor for this purpose. Each drone has its own unique ID that is stored on the server. Using Bluetooth, the drone sends this ID to the smart sensor, which checks against the data in the server to determine whether or not the drone has permission to enter the room. To accomplish this authentication process, we make use of the AllJoyn security platform, which quickly certifies the permission status of the drone. Once this permission has been certified, the door automatically opens, and the drone moves into the room to take photos there. While all of this is happening, the security manager can monitor the whole process on a smartphone, viewing the photographs taken by the drones, as well as the video provided by the CCTV cameras. In addition, when a suspicious situation such as smoke detection occurs, the existing smoke-alert system is activated. The smart sensor transmits smoke data and the video and photo data of CCTV or UAV to the manager. Using this information, the manager can come to a final decision regarding the status of the situation, whether it is an emergency or not.

#### 3.3.3. Emergency Response

**Figure 6:**
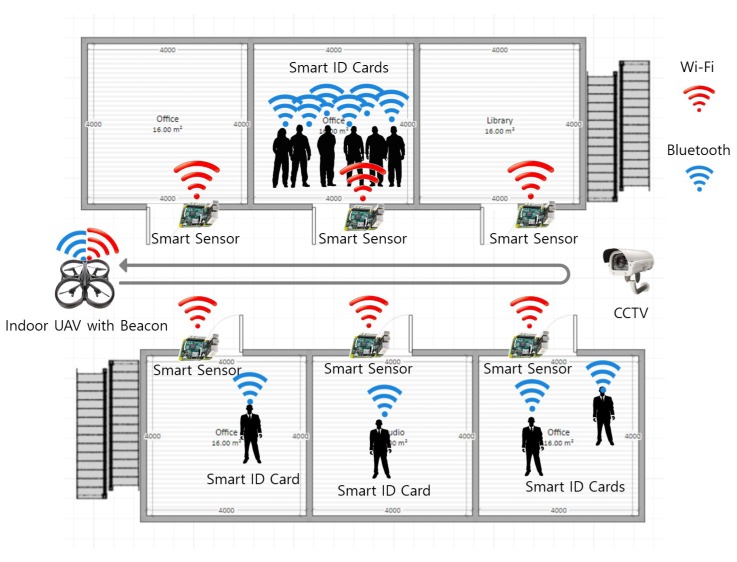
Emergency response process.

In the event that a situation is determined to be an emergency, the building manager begins the emergency response process by sending a message to the control server. In order to prevent malicious interference from external sources or false reports, only managers authenticated by the Alljoyn security platform have the right to begin this process. Once an authenticated message has been received from an authorized source, the control server broadcasts emergency alert messages to everyone in the building and to all smart sensors, drones and other building personnel. In addition, the sprinkler installed in the building is operated by the server. This emergency response process is shown in Figure 6. CCTV and indoor and outdoor drones continue to monitor the situation both inside and outside the building, sending all visual data to the server. Furthermore, in an emergency situation, the smart sensors utilize the beacon signals in the users’ smart ID cards in each room to determine how many users are indoors and transmit the number to the server.

To make the above response possible, the system’s smart sensors are constantly connected to Bluetooth and therefore always capable of receiving a Bluetooth signal from beacons that are attached to or embedded in users’ smart ID cards. In this way, the smart sensors can precisely locate all building occupants at all times. The Bluetooth beacons in the ID cards transmit a constant signal to all the components of the system. This signal includes a time stamp, a TX power setting, an RSSI (dB), a major number and a minor number. The minor number is particularly important, as it is used to provide a unique identification number to each user’s ID card, which is what allows the system to specify exactly where each user is located in the building. The location of an ID card, also called its proximity, can be determined by the strength of the signal as it is received by a sensor. Usually, the Friis model formula [35] is used to calculate this distance. Equation (1) is the Friis transmission equation [35].

(1) $$P_{r}\left(\omega \right)=P_{t}\left(\omega \right)\frac{G_{t}\left(\omega \right)G_{r}\left(\omega \right)\lambda^{2}}{{\left(4\pi r\right)}^{2}}$$

where $P_{t}$ and $P_{r}$ are the transmitted and received powers, respectively, $G_{t}$ and $G_{r}$ are the antenna gains of the receiving and transmitting antenna, respectively, λ is the wavelength at the operating frequency and *r* is the distance between the transmitting and receiving antennas.

By this Friis model formula, the proximity value of beacons *r* is obtained as Equation (2):

(2) $$r=\frac{\lambda }{4\pi }\sqrt{\frac{P_{t}}{P_{r}}G_{t}G_{r}}$$

However, the calculated proximity value *r* has an accuracy of 68% [25]. Therefore, in order to improve the accuracy, we apply the median of the proximity value *r* for a certain period of time to calculate the user’s position.

Therefore, during an emergency situation, the control server informs the occupants in the building of the disaster and, using the proximity data received from their ID cards, determines the optimal emergency route for each person based on his/her current location in the building. In the event that a smart sensor becomes disabled in an emergency situation, by fire, for example, an occupant’s proximity data can also be transmitted by the indoor/outdoor UAVs as they are also constantly receiving that information via Bluetooth.

## 4. Prototype of UAV-EMOR and Use Scenarios

**Figure 7:**
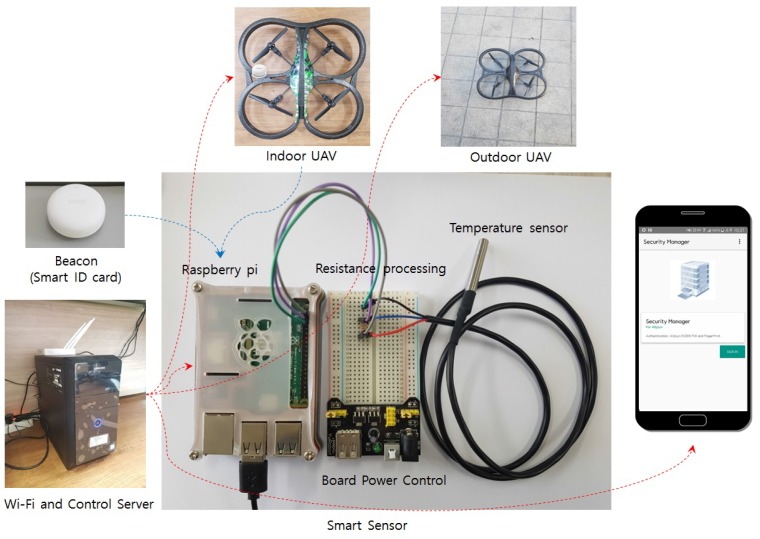
Prototype of UAV-EMOR system.

In order to demonstrate the possibility of our system, we implement prototype for our UAV-EMOR system. In this section, we present the main prototype of our UAV-EMOR system and use scenario. Figure 7 shows the prototypes our UAV-EMOR system. Our prototype consists of commercial AR drones, temperature sensors, Raspberry Pi and a standard control server for implementing functionalities such as fire detection, smart notification and fire response services. Moreover, in order to secure communication within the building, the internal Wi-Fi network was protected by applying the AllJoyn security framework based on P2P, which is an open standard platform for the Internet of Things.

To construct a smart sensor, we combined a conventional DS18B20 sensor for temperature measurement with a single-board computer, the Raspberry Pi. This computer has a built-in input/output pin (General-purpose input/output, GPIO) for hardware control, which is useful for configuring the system to utilize the sensor. The DS18B20 temperature-sensitive drive circuit responds to between the −55 ${}^{\circ }$C and +125 ${}^{\circ }$C range and detects temperatures within −0.5 ${}^{\circ }$C and +0.5 ${}^{\circ }$C. We used the conventional temperature sensor to implement the prototype, but the temperature sensor could be changed to a high-end sensor for real-world use. In addition, on each floor, there are multiple smart sensors with temperature sensors, so the control server can use data from all over the floor to determine the actual temperature. Therefore, the control server can avoid false alarms. If a room sensor registers a high temperature, the control server sends an indoor UAV to the room to check the situation and that will confirm the temperature.

Temperature-sensitive sensors are used for early fire detection. The sensor module for sensing the initial temperature of a fire is connected via the Raspberry Pi GPIO, and everything is linked via the control server. In addition, source coding is performed for the smart sensor using Razbian (a Linux-based OS), which automates data exchange between the control server, sensors and drones and can also automate certain actions, as well. For example, the control server commands the drones to monitor the inside and outside of the building and sends the monitoring results to the security manager so that any abnormal situations can be dealt with promptly.

**Figure 8:**
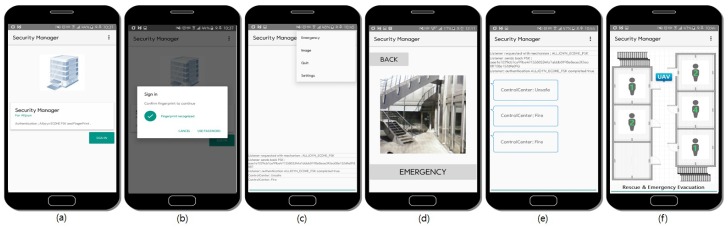
The interface of the building Security Manager app.(**a**) first page of security manager app , (**b**) fingerprint authentication process, (**c**) security manager app in a normal situation, (**d**) an abnormal temperature is detected, (**e**) security manager app in abnormal situation, (**f**) rescue and emergency evacuation mode of security manager app

Figure 8 shows the execution screen of the security manager app, which is used by a security manager to monitor a building. We use a biometric-based authentication method to implement user authentication of the Security Manager app. Therefore, only users who are specifically granted permission can access and control the app. The security manager’s fingerprint is registered on the smartphone, and the only way the app can be accessed and the building monitored is via fingerprint identification. Using this app, the security manager can always check and monitor the inside or outside of the building. Figure 8b shows the user confirmation message, which appears on the screen upon the verification of the security manager’s fingerprint. At the same time, the security manger app generates a PSK-based on the AllJoyn security platform to establish a secure connection with the control server. Our smart building monitoring prototype considers three different statuses: ‘Active’, ‘Unsafe’ and ‘Fire’. In a normal situation, the status of a smart sensor is ‘Active’, which means that conditions are stable. However, if a smart sensor senses a temperature that is outside of the normal range, the status of the sensor is changed to ‘Unsafe’. If the sensor identifies a fire in the building, it changes its status to ‘Fire’.

The Security Manager app receives these status updates on the building through a secure connection between the smart sensors and the control server by using the AllJoyn security platform. When the security manager receives notice of an emergency situation on the Security Manager app, he/she can check the extent of the danger and the size of any fires through real-time video and photos taken by indoor and outdoor drones. As shown in Figure 8d, if an abnormal temperature is detected, the control server registers the ‘Unsafe’ status, then transmits this status information to all the smart sensors in the building, to the indoor and outdoor drones and to the security manger smartphone. Once the security manager checks the detailed information of a fire situation, he/she can declare an emergency command by pressing the ‘Emergency’ button, which will set in motion the fire emergency response procedure.

## 5. Performance Evaluation

**Figure 9:**
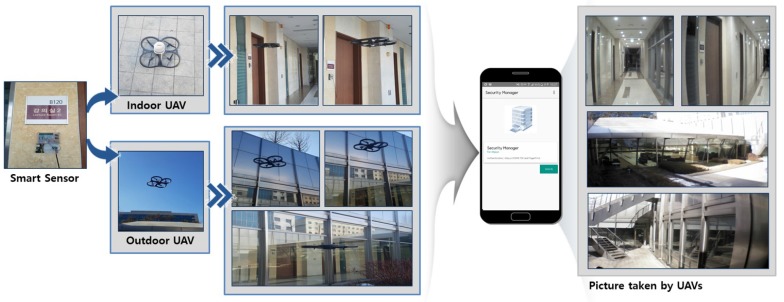
The experimental flow of the day-to-day monitoring process.

**Figure 10:**
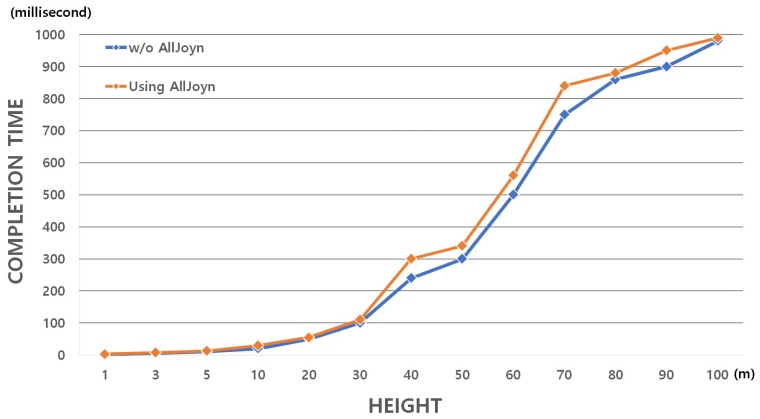
The communication and computation overhead according to flight altitude.

In order to evaluate the performance of our UAV-EMOR system protocol, we implemented our day-to-day monitoring protocol on a commercially available augmented reality drone (AR.Drone) 2.0 for both indoor and outdoor monitoring. The AR.Drone 2.0 is a quad-copter equipped with front and ground cameras and Wi-Fi (2.4 GHz). It has a 1-GHz 32-bit ARM cortex A8 CPU and a 1 GB RAM. The main board of the AR.Drone 2.0 runs the BusyBox-based GNU/Linux distribution with the 2.6.32 kernel. We also used a personal computer with Linux OS as the control server and Raspberry Pi as a smart sensor. Figure 9 shows the experimental flow of the day to day monitoring protocol. In this experiment, we mainly evaluated the performance of the indoor/outdoor UAV and smart sensors during execution of the protocol, as they are resource-constrained devices. We measured the completion time of the protocol for day-to-day monitoring to evaluate the communication overhead according to flight altitude. For evaluating the communication overhead, we placed the server on the ground and periodically increased the drone’s flying altitude, measuring the communication time between the UAV and the server at each altitude. The parameters used in the simulation are shown in Table 2. We measured the communication time between the UAV and the server under two different circumstances: (1) communication secured by an encryption algorithm, AES (Advanced Encryption Standard) on the AllJoyn Security platform, and (2) unsecured communication without using the encryption algorithm on the AllJoyn security platform. The communication time was measured from the time that a ping was sent from the server to the time that the drone’s response ping was received back at the server. Figure 10 shows the communication overhead according to the distance between the UAV and the server. In Figure 10, we can see that, generally, the communication overhead increases as the distance between the UAV and the server increases. However, it can also be seen that the communication overhead is not significantly increased by the use of an encryption algorithm for a secure communication, as represented on the graph by the orange line.

Moreover, in order to evaluate the computational overhead of the AR.Drone 2.0 (which we used as our indoor/outdoor UAV), we measured the encryption/decryption time of messages or images to complete the monitoring mission of UAV. Table 2 shows the time required to execute encryption and decryption algorithms for completion of a mission according to the size of the messages or images. When the size of the text message for transmission was 128 bytes, the UAV with our protocol took 1.1 ms and 0.9 ms to complete the protocol with encryption and decryption, respectively. Even for a large text size such as 10,240 byte, the UAV with our protocol encrypted and decrypted the message relatively quickly. It took only 180 ms to perform the encryption or decryption operation. Generally, images take longer to encrypt and decrypt than text messages do. However, our system is still relatively fast in this regard. It took the UAV 15 ms to encrypt/decrypt 473 bytes (one or two images). For the secure transmission of 10,703 bytes (about six or seven images), the UAV took 306 ms.

Furthermore, in order to evaluate the computational overhead of the smart sensor, we measured the cryptographic operation time such as the encryption or decryption time of messages to securely communicate with the UAV. Table 3 shows the computation time required to execute encryption and decryption algorithms for secure communication to the size of the messages. When the size of the transmitted text message was 256 bytes, the smart sensor took 1.79 ms and 1.77 ms to complete the protocol with encryption and decryption, respectively. Even for a large text size such as 10,240 byte, the smart sensor encrypted and decrypted the message relatively quickly. It took only 164 ms to perform the encryption or decryption operation.

## 6. Discussion

### 6.1. Position Accuracy

The distance measurement using the beacon is based on the received Bluetooth signal strength. However, if signal interference due to surrounding obstacles occurs, the measured distance data may be inaccurate. Therefore, the proximity value of a beacon calculated using the Friis model formula has generally low accuracy such as 68%, due to frequent signal interference [24,36]. To improve the accuracy, Choi et al. [37] used the average value after storing the distance calculation results for 30 s. However, the method of taking the average value shows higher accuracy only when the experiment is performed by constructing the optimal environment without signal interference. If a large distance error is transmitted due to Bluetooth signal interference, the large error value, i.e., the outlier, affects the average value.

In order to reduce the effect of outliers and improve the position accuracy measured by the beacon, we propose to apply the median of the proximity value *r* for a certain period of time to measure the occupant’s precise location. In order to evaluate the accuracy of our system’s proximity data, we performed the experiment using raw values, the average of all values and median values in a variety of emergency scenarios. For our experiments, it is assumed that the building is fully outfitted with smart sensors and that each building occupant carries a beacon-embedded smart ID card that is used to locate the occupant during an emergency.

**Figure 11:**
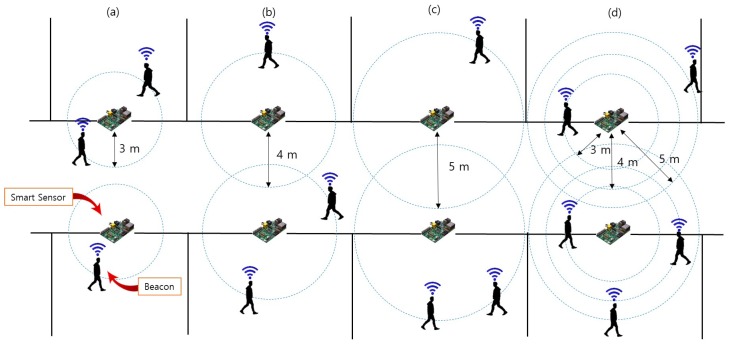
The experimental setting used for localization accuracy evaluation. Participant walks in a (**a**) 3 m radius, (**b**) 4 m radius, (**c**) 5 m radius, (**d**) 3–5 m radius.

Figure 11 shows four experimental conditions. We attached smart sensors to the front of the door in each room, and the installation radius between sensors was 3–5 m. Each experiment participant carried a beacon and was instructed to walk in a radius of 3 m, 4 m then 5 m away from a smart sensor. Figure 11a–c indicates that a user with a beacon is moving or standing at a distance of 3 m, 4 m and 5 m from the smart sensor, respectively. Figure 11d shows the experimental environment in which the participants were allowed to either remain in one location or to freely change their location while remaining within the 3–5 m radius from the smart sensor.

**Figure 12:**
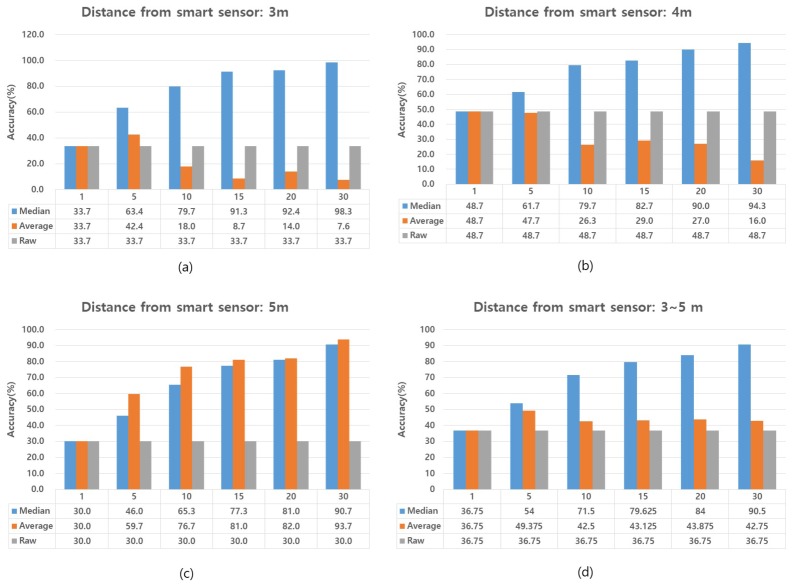
Accuracy of proximity calculations. The interval between smart sensor and beacon is (**a**) 3 m, (**b**) 4 m, (**c**) 5 m, (**d**) 3–5 m.

Figure 12 shows the results of our position accuracy calculations. To evaluate position accuracy, we used the following three methods: the raw method [24], the average method [37] and the median method. The raw method accepts the proximity value calculated as the distance of the beacon from a single smart sensor. The average method and the median method both refer to methods in which the average or median of multiple proximities is obtained by calculating the location of the beacon over a set period of time. In order to evaluate the accuracy of these methods, we tested different periods of time for collecting the proximity data. These set periods of proximity data collection were set to 1 s, 5 s, 10 s, 15 s, 20 s and 30 s.

One challenge to calculating precise locations with beacons is that smart sensors are affected by where a user holds the beacon, i.e., on the upper or lower body. This is partly responsible for the sometimes wide variance in proximity data that smart sensors receive from beacons. In our study, the goal of position recognition is to locate building occupants and inform them of appropriate evacuation routes in an emergency situation. For this purpose, we considered a small location range of up to 1 m acceptable. Accordingly, we set a tolerance threshold value of 50 cm on the data received from the smart sensor. That is, if the smart sensor calculates the position of the user as being 3 m away, we regard the user’s correct position as a range from 2.5–3.5 m.

Experimental results show that the accuracy of proximity data provided by systems that use beacons is significantly low (33.7%, 48.7% and 30%). This is because beacons broadcast inconsistent proximity data. Most of the data broadcasts may be correct, but on occasion, they may also broadcast a highly incorrect distance. For example, if a user is 3 m away from a smart sensor, the raw method may read an incorrect broadcast from a beacon and calculate that the user is at a distance of 12 m. Likewise, the inaccuracy of beacon broadcasts also adversely affects the data calculated using the average method. At distances of 3–4 m, the average method shows decreasing accuracy as time for collecting the proximity data increases; see Figure 11a,b. For these reasons, neither the raw method, nor the average method are suitable for the calculation of precise location using beacons. In contrast to those methods, the median method provided a significantly high level of accuracy. This accuracy was also sustained over long periods of collection time for the proximity data. At all distances, 3 m, 4 m and 5 m, the proximity data gathered in a 30-s period and calculated using the median method showed an accuracy of more than 90%. Thus, in our UAV-EMOR system, we program smart sensors to collect proximity data from a beacon for 30-s periods, then to calculate the median proximity value of that data and send it to the server as the user’s location.

### 6.2. Privacy Considerations

Recently, many studies related to location privacy have gained much attention, because users may want to use location-based services without revealing their location. Location privacy in wireless sensor networks including IoT environments may be classified into content privacy and contextual (e.g., the traffic) privacy. Several approaches for protecting content-oriented privacy have been designed in location tracking systems, which can decide the users’ positions for location-based services [38,39,40]. Spreitzer et al. [38] utilize a location broker residing at the middleware layer to hide users’ positions. Gruteser et al. [39] agitate the sensed location information by using the k-anonymity criterion. Al-Muhtadi et al. [40] presented the location privacy protection method through a mist router and a handle-based virtual circuit routing protocol. In addition, to protect the receiver’s (i.e., the sink) location, privacy is important, because the receiver collects data from all sensors. Deng et al. [41] first introduced the issue of how to hide the location of the sink (e.g., the base station) and presented techniques of multi-path routing and fake message injection. Jian et al. [42] proposed a location-privacy routing protocol incorporating fake packet injection.

In our UAV-EMOR system, the beacon sensors are used to implement rescue and evacuation functionality. Using Bluetooth technology, such beacons constantly transmit the unique ID and real-time location of the occupants to all smart sensors and indoor-UAVs. Since the building management system can guide the occupants to the evacuation route based on these real-time location data and unique ID, it is useful for evacuating occupants in an emergency situation. The control server of the UAV-EMOR system allows smart sensors and indoor UAVs to enable Bluetooth and receive beacon signals from users only when the emergency situation happens. The system may be connected at all times, but the control server does not collect or store the users’ location information at normal times because it has no need to ask for the information. Still, the use of location information can introduce potentially unresolved privacy concerns [43]. Therefore, we need to consider the location privacy issue; simply put, we can make the security policy for our UAV-EMOR to update the beacon ID periodically. To more actively protect the location privacy, we can apply the above mentioned studies about location privacy protection in wireless sensor networks to the UAV-EMOR system.

## 7. Conclusions

In this paper, we have presented a proof of concept prototype of a monitoring and emergency response method that we call the UAV-EMOR system that is intended to address the weaknesses common in such smart systems, specifically limited monitoring range, inaccuracy of proximity data and data security. In order to enhance the real-time monitoring range, we present an approach that incorporates indoor/outdoor UAVs in coordination with smart sensors; to improve the accuracy of data received from beacons, we introduce the median method of proximity data calculation; and to address security issues, we use the AllJoyn security platform to provide strong encryption for all communications between all components used in the system.

## Figures and Tables

**Table 1:** Comparison of related works. EMOR, Emergency Monitoring and Response.

	Source		Types of Functionalities				Service Area	User Interface
	Sensor	UAV	Monitoring	Alarm	Rescue	Security		
UAV-EMOR (Our System)	O	O	O	O	O	O	Building, Outside	Smartphone App
[2]	O			O	O		Building	Smartphone App
[3]	O		O	O	O		Building	
[4]	O		O	O	O		Building	
[5]	O		O				Building	
[6]	O		O	O	O		Building	Smartphone App
[7]	O	O	O				Nature	
[9]	O	O	O				Nature	
[10]	O		O	O			Nature	Web
[11]	O	O	O				River	

**Table 2:** Computation time on UAVs.

	Text					Image				
Message Size (Byte)	32	128	512	2048	10,240	473	824	2453	8530	10,703
Encryption (ms)	0.6	1.1	5	40	180	15	36.5	106	220.5	306
Decryption (ms)	0.6	0.9	5	40	180	15	36.5	106	220.5	306

**Table 3:** Computation time on a smart sensor.

	Text									
Message Size (Byte)	16	32	64	128	256	512	1024	2048	4096	10,240
Encryption (ms)	0.31	0.5	0.7	0.86	1.79	4.1	13.4	36	71	164
Decryption (ms)	0.31	0.49	0.69	0.86	1.77	4	13.3	36	70	164
